# Classifying Degraded Modern Polymeric Museum Artefacts by Their Smell

**DOI:** 10.1002/anie.201712278

**Published:** 2018-03-02

**Authors:** Katherine Curran, Mark Underhill, Josep Grau‐Bové, Tom Fearn, Lorraine T. Gibson, Matija Strlič

**Affiliations:** ^1^ UCL Institute for Sustainable Heritage University College London 14 Upper Woburn Place London WC1 H 0NN UK; ^2^ Department of Statistical Science University College London Gower Street London WC1E 6BT UK; ^3^ Department of Pure and Applied Chemistry University of Strathclyde Thomas Graham Building, 295 Cathedral Street Glasgow G1 1 XL UK

**Keywords:** gas chromatography, heritage science, mass spectrometry, plastics conservation, volatile organic compound analysis

## Abstract

The use of VOC analysis to diagnose degradation in modern polymeric museum artefacts is reported. Volatile organic compound (VOC) analysis is a successful method for diagnosing medical conditions but to date has found little application in museums. Modern polymers are increasingly found in museum collections but pose serious conservation difficulties owing to unstable and widely varying formulations. Solid‐phase microextraction gas chromatography/mass spectrometry and linear discriminant analysis were used to classify samples according to the length of time they had been artificially degraded. Accuracies in classification of 50–83 % were obtained after validation with separate test sets. The method was applied to three artefacts from collections at Tate to detect evidence of degradation. This approach could be used for any material in heritage collections and more widely in the field of polymer degradation.

Volatile organic compound (VOC) analysis has been researched extensively for disease diagnosis via detection of chemical markers.^1^, ^2^ It has been used to diagnose lung cancer and Alzheimer's disease, among many others.^1^ Key advantages of VOC analysis are that it provides insight into complex and varied chemistries (for example, disease pathology in different individuals) and that it is non‐invasive (for example, breath analysis). These advantages are relevant within cultural heritage where degradation of a museum artefact represents a complex and varied chemical system. Non‐invasive analysis is favoured in heritage to minimise damage to valuable artefacts. VOC analysis has previously been explored in the context of historic paper, and volatile degradation markers were linked to material properties such as degree of polymerisation.^3^ In this work we introduce VOC analysis as a method for diagnosing degradation in modern polymeric artefacts, such as plastics. Previous publications by the authors and others have identified VOCs characteristic of particular polymers, such as plasticisers, monomer residues, and oxidation products.^4^, ^5^, ^6^ However, the use of VOC analysis to classify such artefacts according to their degradation remains unexplored.

Modern polymers are present in increasingly large numbers in heritage collections. Found in modern art and design collections (Figure 1), 20th century social history objects and archival materials such as celluloid film, they form extremely valuable artefacts essential to protect for future generations. However their conservation presents serious challenges owing to inherent instability and a very wide range of materials in collections, incorporating different polymers and additives.^7^ There is thus an urgent need for new methods to detect evidence of degradation in such objects in collections.

**Figure 1:**
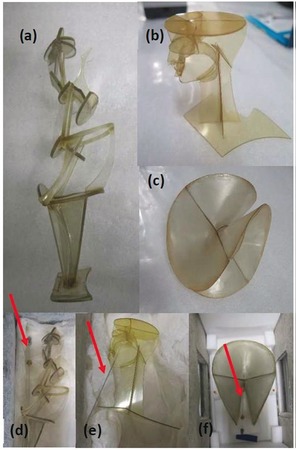
Tate plastics‐based objects analysed as part of this research, as packed in August 2017. a) Naum Gabo, *Model for the statue of Aphrodite in the ballet “La Chatte”* 1927 (Tate T02242). The Work of Naum Gabo © Nina & Graham Williams/Tate, London 2017. b) Antoine Pevsner, *Head* 1923–24 (Tate T02241). ©ADAGP, Paris, and DACS, London, 2017. c) Naum Gabo, *Model for Spheric Theme* c.1937 (Tate T02173). The Work of Naum Gabo © Nina & Graham Williams/Tate, London 2017. d),e),f) The same objects with the SPME fibre in place (marked by arrow).

The first use of VOC analysis is reported as a classification tool to study degradation in modern polymeric objects in museums. Solid‐phase microextraction gas chromatography mass spectrometry (SPME‐GC/MS)^8^ was used to detect VOCs from 50 mg samples of 96 modern polymeric objects. These included objects with a range of formulations dating from between 1920s–2000s with base polymers known to be problematic in museums: cellulose nitrate (CN), cellulose esters such as cellulose acetate (CA) and cellulose propionate (CP), polyurethane (PUR) foams and poly(vinyl chloride) (PVC),^9^ and other materials found in collections such as polystyrene (PS) and polyethylene (PE) based on museum surveys.^10^, ^11^, ^12^, ^13^ A full list of objects is provided in the Supporting Information. Pieces from 25 objects, including multiple examples of each polymer type, were degraded at 80 °C and 65 % relative humidity (RH) for 2, 4, 6, 8, or 10 weeks. 50 mg samples from these pieces were also analysed using SPME‐GC/MS, making the total number of samples 211. The samples capture some of the compositional variation in collections, although a more extensive sample set would be more representative. Concentrations of detected VOCs were not calculated, however peak areas of relevant VOCs were calculated and weighted using a standard to ensure inter‐day repeatability and can thus be compared quantitatively. After each time period, samples were removed from the degradation chamber for SPME‐GC/MS at room temperature, to be relevant to a museum context. An on‐site experiment at Tate used SPME‐GC/MS to analyse VOC emissions from three modern polymeric objects: *Model for the statue of Aphrodite in the ballet “La Chatte”* (1927) composed of CA and *Head* (1923–24) (CN) by Antoine Pevsner and *Model for Spheric Theme* (c. 1937) (CA) by Naum Gabo (Figure 1).

Detected VOCs give insight into the composition and ongoing degradation processes of the objects. Analysis of 25 CN samples shows an increase in furfural emissions over time (Figure 2 a). Furfural is a product of the acid‐catalysed hydrolysis of cellulose^14^ and our results show that VOC analysis can be used to study this process in CN. Camphor was detected from all CN samples, this was expected as it was a common plasticiser for CN.^15^ Other related compounds, for example camphene and campholenal, were also detected.

**Figure 2:**
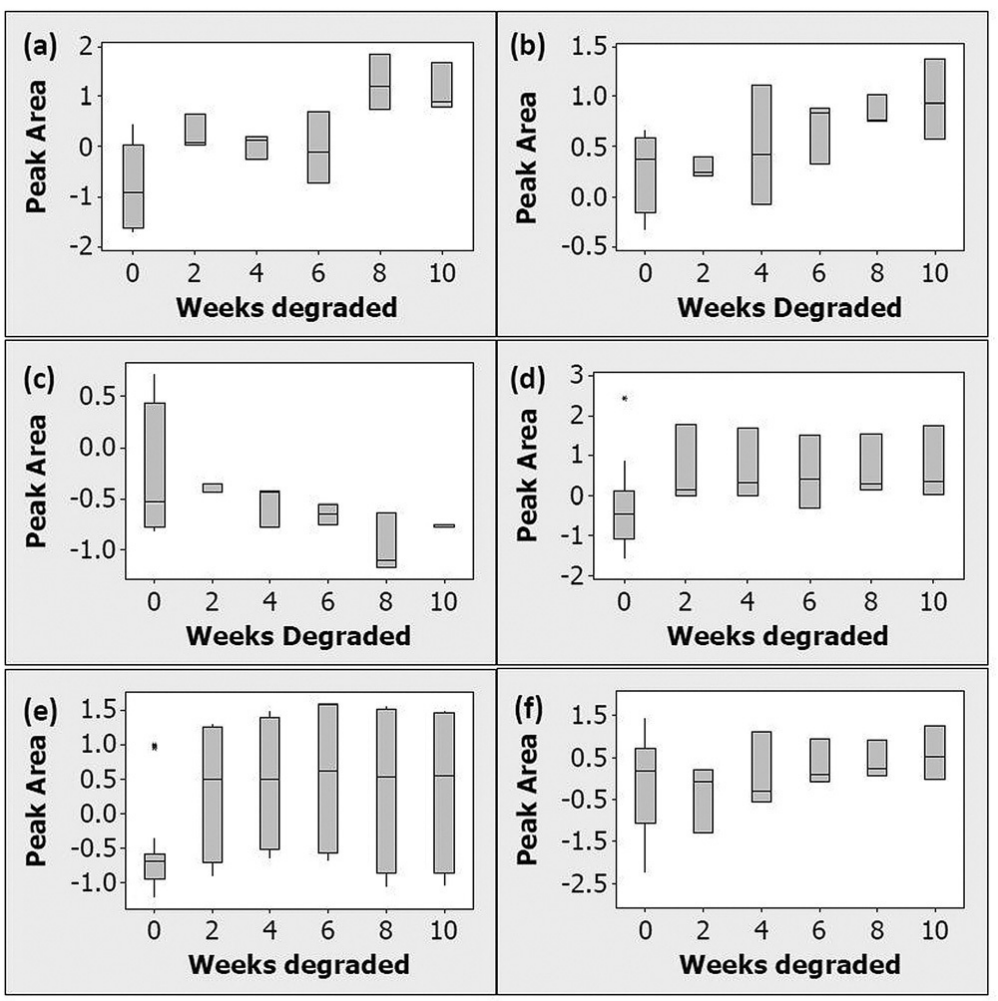
Boxplots showing changes in analysed VOC emissions from modern polymeric samples after 0, 2, 4, 6, 8, or 10 weeks of degradation at 80 °C and 65 % RH. a) Furfural emissions from 25 CN samples, b) propanoic acid emissions from 19 CP samples, c) dimethyl phthalate emissions from 19 CP samples, (d) 2‐ethylhexanol emissions from 39 PVC samples, e) pentanal emissions from 36 PUR samples, and f) *cis*‐β‐methylstyrene emissions from 30 PS samples. Peak areas were weighted using a standard solution run several times on each day of analysis, log‐transformed, and normalised to a mean of 0 and standard deviation of 1.

Propanoic acid (PA) emissions increased over time for 19 samples of CP, while emissions of dimethyl phthalate plasticiser decreased (Figure 2 b,c). PA is formed from hydrolysis of side groups on CP; phthalate plasticiser loss from cellulose ester objects in museums is a known degradation process leading to brittleness and cracking.^16^ We show that both processes can be tracked using VOC analysis. 2‐Ethylhexanol (EH) was detected from 39 PVC samples and found to increase after 2 weeks of thermal ageing, remaining relatively constant for the remaining time (Figure 2 d). EH is a known degradation product of bis(2‐ethylhexyl)phthalate (DEHP), a common plasticiser in PVC objects.^17^ PUR samples were found to emit 5‐ethenyldihydro‐5‐methyl‐2(3*H*)‐furanone and aldehydes, including pentanal (Figure 2 e), hexanal, and benzaldehyde. Aldehyde formation occurs owing to PUR oxidation via formation of a macroalkoxy radical,^18^ while furanones are known emissions from thermally degraded PUR‐based magnetic tape.^19^ However, clear trends in emissions from PUR samples could not be seen.

For PS, known thermo‐oxidative degradation products were detected, including acetophenone, benzaldehyde, and *cis*‐β‐methylstyrene.^20^ It was also difficult to detect trends in these emissions (see the example of *cis*‐β‐methylstyrene in Figure 2 f). The main VOC emissions from PE objects were hydrocarbons such as decane and undecane, which are unlikely to be degradation products.

While our results show that VOC analysis can be used to monitor ongoing degradation processes, clear trends cannot always be seen using single VOCs. We therefore used combinations of VOCs to classify samples based on degradation. For each polymer type, samples were divided into two classes:

- Class 1: samples artificially degraded for 0–4 weeks
- Class 2: samples artificially degraded for 6–10 weeks

This is an unbiased classification in which we are confident that one class has been exposed to more degrading conditions than the other and each class had similar sample numbers. The classes do not correspond to evidence of physical damage, for example discolouration or cracking, although in some cases physical damage was seen to increase as objects moved from Class 1 to Class 2. Further work would be needed to explore such a classification. Linear discriminant analysis (LDA), a well‐known method for classification using VOC data,^1^, ^21^ was used to assess whether detected VOCs can be used to accurately assign samples to a class and thus to distinguish between samples that were more or less degraded. LDA develops predictive functions based on linear combinations of analysed VOC emissions. A two‐tailed t‐test first identified VOCs that differed significantly between classes. Multiple combinations of VOCs were tested to identify which ones gave the most accurate classifications. Results are shown in Table 1. Validation accuracy (VA) was used to assess the success of classification. Validation was performed by dividing samples of a particular polymer into a training set and a test set. Sets were chosen so that no samples in the test set came from the same object as samples in the training set. Owing to the number of samples, test sets were smaller than training sets, in all but one case test sets consisted of 6 samples, all taken from one object and aged for different lengths of time that is, 0, 2, 4, 6, 8, and 10 weeks. For each polymer type, 3 to 6 separate test sets were used to validate the model. VA is the average of the percentages of samples from separate test sets that were classified accurately. Further detail about data analysis is found in the Experimental Section and in the Supporting Information.

**Table 1:** Results of classification of modern polymeric samples according to length of exposure to artificial degradation using detected VOC emissions and linear discriminant analysis.

Polymer	Classification accuracy^[a]^	Validation accuracy^[b]^	No. of samples^[c]^	No. of test sets	VOCs used for prediction
CN	93	83	19	3	furfural, terpene1,^[d]^ camphene, campholenal
PUR	87	79	30	4	camphor, phenol, pentanal, 3,5‐dimethyloctane, styrene
CP	82	78	13	3	propanoic acid, dimethyl phthalate
PS	77	62	24	4	*cis*‐β‐methylstyrene, acetophenone, (1‐methylethyl)‐benzene
PE	63	53	42	6	decane, camphor
PVC	82	50	33	3	hexanal, 2‐ethylhexanol, limonene

[a] Average of accuracy of initial classification, excluding separate test sets. [b] Average of accuracy of validation using 3–6 different separate test sets. [c] No. of samples used to build predictive model. [d] The compound named as “terpene1” was identified as either 3‐carene or β‐terpinene.

Classification of CN samples achieved a VA of 83 %. VOCs that produced the most accurate classification were furfural and camphor derivatives such as camphene, campholenal, and a terpene identified as either 3‐carene or β‐terpinene. Furfural emissions were higher in Class 2 than in Class 1, while emissions of the other VOCs were lower, corresponding to CN hydrolysis and loss of camphor plasticiser and related compounds.

For PUR, the most accurate classification used five VOCs: camphor, phenol, pentanal, styrene, and 3,5‐dimethyloctane, with a VA of 79 %. Samples in Class 2 had higher pentanal emissions than those in Class 1, which is most likely due to oxidative formation of aldehydic groups.^18^ Class 1 had higher emissions of the other four VOCs, indicating that they are more likely to be found in less degraded PUR samples. Phenol may have originated from catalysts used to make PUR.^22^ The origins of the other VOCs is unknown. A VA of 78 % was achieved for CP samples, using PA and dimethyl phthalate. As stated, both VOCs correspond to known degradation processes. It was not possible to include CA in the classification as aged samples from only one object were analysed, meaning that no separate test sets could be used.

The VOCs that classified PS samples most accurately (62 %) were (1‐methylethyl)‐benzene, *cis*‐β‐methylstyrene, and acetophenone. Samples in Class 2 had higher emissions of the oxidation products *cis*‐β‐methylstyrene and acetophenone compared with Class 1 and lower emissions of (1‐methylethyl)benzene. This corresponds to results from previous work on recycled and virgin PS.^23^ (1‐methylethyl)benzene was found in greater abundance in virgin PS than oxidation products such as acetophenone and benzaldehyde, indicating that (1‐methylethyl)benzene may be a manufacturing residue lost during use, not a degradation product formed over time.

Classification of PE and PVC samples was not successful; VAs were only 53 % and 50 %, respectively. Known products of PE oxidative degradation, for example carboxylic acids and ketones, were not detected, suggesting that degradation conditions used were not extreme/long enough to induce degradation.^24^ Discolouration of PVC samples was observed, this can be due to the unzipping of PVC, forming conjugated double bonds via HCl release.^17^ HCl is not detectable via our SPME‐GC/MS method so this process could not be studied. In the case of both PE and PVC, light ageing may result in the emission of more detectable volatile degradation products and would warrant further study.

The method was applied to three modern polymeric artefacts from Tate. Artefacts were classified using similar VOCs to those that previously gave the highest VAs. To account for differences in the mass of the artefacts relative to the samples and in the volume of the headspace, ratios of VOCs were used rather than peak areas. Classification of the CA objects *Model for the statue of Aphrodite in the ballet “La Chatte”* and *Model for spheric theme* was done using the classification developed for CP samples (Table 1). CP and CA are very chemically similar and organic acids and phthalate plasticisers are key volatile degradation products of both materials. It was not possible to access a CP object at Tate. Using the ratio of acetic acid to dimethyl phthalate both artefacts were classified as members of Class 1. This means that their VOC profile is similar to the samples degraded for 0–4 weeks, rather than for 6–10 weeks. In samples, as the period of artificial degradation progressed, the ratio of acid to phthalate increased due to ester sidechain hydrolysis and plasticiser loss. The CA objects from Tate seem to be at an earlier stage of this process, with relatively low acid to phthalate ratios.

A similar approach was taken for *Head* (CN). Classification was performed using ratios of furfural/terpene, furfural/camphene and furfural/campholenal. *Head* was classified as part of Class 2 that is, to be at a more advanced stage of degradation. For artificially degraded samples, all three ratios increase as ageing time increases, due to degradation of the CN polymer and loss of camphor plasticiser derivatives. *Head* seems to be at a later stage of this process, with high emissions of furfural relative to the other VOCs. There is thus more evidence of chemical degradation in *Head* than in the other artefacts, which suggests that it represents a priority in terms of preventive conservation measures and further analysis. A limitation of this work is that relative rates of plasticiser loss and hydrolysis induced by thermal ageing at 80 °C are likely not the same as relative rates in museum conditions. Further work to develop a classification based wholly on naturally aged samples would help address this.

In conclusion, VOC analysis is a promising and novel method for diagnosing degradation in modern polymeric artefacts. Changes in degradation markers over a period of artificial degradation were studied and samples were classified according to the length of degradation time, based on emitted VOCs. For CN, PUR, CP, and PS, between 62–83 % accuracy was achieved after validation. For PE and PVC, classifications were poor. The method was applied to artefacts from Tate, and ratios of key VOCs used to suggest which artefacts may be conservation priorities. The work introduces a new method that gives valuable chemical information to study degradation of modern polymeric artefacts in museums with potential to be a non‐invasive method for diagnosing degradation in any historic material. Further work is ongoing.

## Experimental Section

SPME‐GC/MS was carried out according to a published method and is described in the Supporting Information.^5^ Samples were from the Historic Plastic Reference Collection at the UCL Institute for Sustainable Heritage, the SamCo collection from the Preservation of Plastic Artefacts (POPART) project,^25^ and the RESINKIT company, Woonsocket, RI, USA. Work at Tate was carried out by placing an exposed SPME fibre in the storage container of each object (Figure 1) for 1 week at room temperature. SPME‐GC/MS analysis used a DVB/CAR/PDMS SPME fibre (50/30 μm) (Supelco, 57298‐U), a PerkinElmer Clarus 500 gas chromatograph equipped with a Combipal PAL System (CTC Analytics) autosampler coupled to a PerkinElmer Clarus 560D mass spectrometer. Peak identification used the NIST 2005 Mass Spectra Library V2.1. Chromatographic data was processed using XCMS Online from the Scripps Center for Metabolomics.^26^ Linear Discriminant Analysis was carried out using IBM SPSS Statistics 22. Further information about data processing is given in the Supporting Information. Polymer types were identified by attenuated total reflectance Fourier transform infrared spectroscopy (ATR‐FTIR) for samples with an ATR Platinum Diamond single‐reflection module no. CFBFA32D and using diffuse reflectance Fourier Transform infrared spectroscopy (DRIFTS) with accessory ID 30GradFocRefl no. 7F992C2D for artefacts at Tate using a Bruker Alpha FTIR Spectrometer. 64 scans were collected over the wavenumber range 4000–375 cm^−1^ with a resolution of 4 cm^−1^.

## Conflict of interest

The authors declare no conflict of interest.

## Supporting information

As a service to our authors and readers, this journal provides supporting information supplied by the authors. Such materials are peer reviewed and may be re‐organized for online delivery, but are not copy‐edited or typeset. Technical support issues arising from supporting information (other than missing files) should be addressed to the authors.

Supplementary
