# Supplementary material for: Classifying Degraded Modern Polymeric Museum Artefacts by Their Smell
Source: Angew Chem Int Ed Engl. 2018 Mar 2;57(25):7336–40. doi: 10.1002/anie.201712278 (PMC6032996; doi:10.1002/anie.201712278)
Supplement: Supplementary file 1 — Supplementary [file ANIE-57-7336-s001.pdf]

Angewandte Chemie
Eine Zeitschrift der Gesellschaft Deutscher Chemiker

Supporting Information

Classifying Degraded Modern Polymeric Museum Artefacts by Their Smell

Katherine Curran, Mark Underhill, Josep Grau-Bové, Tom Fearn, Lorraine T. Gibson, and Matija Strlič**

anie_201712278_sm_miscellaneous_information.pdf

Table of Contents

Table of Contents 1

1 Introduction 2

2 SPME-GC/MS analysis of modern polymeric samples 2

3 Data processing using XCMS Online 5

4 Microsoft Excel 2016 data processing workflow 8

5 Linear Discriminant Analysis in IBM SPSS Statistics 22 9

6 Data from analysis of VOC emissions from modern polymeric samples 12

6.1 Data from analysis of VOC emissions from Cellulose propionate 12

6.2 Data from analysis of VOC emissions from Cellulose nitrate 12

6.3 Data from analysis of VOC emissions from Polyethylene 13

6.4 Data from analysis of VOC emissions from Polystyrene 14

6.5 Data from analysis of VOC emissions from Polyurethane 15

6.6 Data from analysis of VOC emissions from Poly(vinyl chloride) 15

7 Analysis at Tate 16

8 Bibliography 17

1 Introduction

The Supplementary Information provides details of the objects analysed, the SPME-GC/MS method used, the data processing methods and the statistical analysis.

2 SPME-GC/MS analysis of modern polymeric samples

The modern polymeric objects analysed for this research and their polymer types are shown in Table 1. The objects were chosen to represent the range of material types found in museum collections. The objects from which pieces were taken and degraded for 0,2,4,6,8 and 10 weeks at 80 °C and 65% relative humidity (RH) are also shown. The degraded pieces were also sampled as described below. The total number of samples analysed was 211.

Table 1. Modern polymeric objects analysed during this work.

**Polymer type**	**Object no.**	**Object description**
**Cellulose Acetate**		
1[a]	HS91	Brush
2	HS251	White object
3	HS252	Yellow object
4	HS304	Film
5	HS331	Pale brown object
**Cellulose Acetate Derivatives**		
6[a]	HS34 (CP)	Resinkit
7[a]	HS35 (CP)	Resinkit
8[a]	HS45 (CP)	Resinkit
9	HS166 (CP)	Black frame
10	HS250 (CAB)	Orange object
**Cellulose Nitrate**		
11[a]	HS2	Jewellery box
12[a]	HS3	Jewellery box
13[a]	HS104	Vanity set
14	HS232	Cream box
15	HS248	Ruler
16	HS249	Ruler
17	HS266	Brown box
18	HS270	Comb
19	HS271	Cigarette case
20	HS311	Transparent object
**Poly(vinyl chloride)**		
21	HS5	Doll
22[a]	HS33	Doll
23[a]	HS61	Resinkit
24[a]	HS62	Resinkit
25	HS112	Doll

26	HS114	Doll
27	HS115	Doll
28	HS116	Doll
29	HS121	Barbie doll head
30	HS122	Barbie doll head
31	HS123	Barbie doll head
32	HS124	Ken Doll head
33	HS125	Ken Doll head
34	HS126	Ken Doll head
35	HS127	Ken Doll head
36	HS129	Barbie doll head
37	HS153	White/grey object
38	HS157	Transparent object
39	HS170	Glasses frame
40	HS172	Glasses frame
41	HS173	Glasses frame
42	HS174	Glasses frame
43	HS175	Glasses frame
44	HS176	Glasses frame
45	HS321	White object
**Polyurethane**		
46	HS69	Cream ResinKit
47[a]	HS85	White object
48[a]	HS86	White object
49[a]	HS89	Foam toy
50[a]	HS90	Foam toy
51	HS201	Artificial leather
52	HS202	Artificial leather
53	HS206	Foam
54	HS244	Foam
55	HS245	Foam
56	HS257	Textile
57	HS258	Textile
58	HS316	Foam
59	HS317	Foam
60	HS328	Transparent sample
61	HS329	Transparent sample
**Polystyrene**		
62	HS4	Pink box
63[a]	HS14	Red holder
64	HS25	Box
65	HS32	White ball
66[a]	HS36	Resinkit
67[a]	HS37	Resinkit
68	HS38	ResinKit
69	HS118	Necklace

70	HS119	Necklace
71	HS141	Doll head
72	HS145	Green cup
73	HS168	Record duster
74	HS261	Transparent object
75	HS300	Black object
76	HS312	Foam
77	HS322	Transparent object
**Polyethylene**		
78	HS1	White lid
79[a]	HS13	Doll
80	HS15	Doll
81[a]	HS24	Doll
82[a]	HS28	Black sheet
83[a]	HS31	Toy car
84	HS55	Resinkit
85[a]	HS56	Resinkit
86[a]	HS57	Resinkit
87	HS80	Resinkit
88	HS113	Toy car
89	HS117	Red squeezer
90	HS120	White pot
91	HS128	White pot
92	HS130	Yellow pot
93	HS147	Green bottle lid
94	HS208	Transparent bag
95	HS263	Transparent object
96	HS299	White object

[a]Objects from which pieces were taken and artificially degraded for 0,2,4,6,8 and 10 weeks at 80 °C and 65% RH. The degraded pieces were also sampled and analysed using SPME-GC/MS.

The development of the solid-phase microextraction gas chromatography/mass spectrometry (SPME-GC/MS) method has been described elsewhere.[1] Objects were sampled by grinding using an Everise Rotary Tool (Code: N60GR) to collect 50 mg samples. It had previously been found that grinding homogenised the sample surface area and led to more repeatable analysis. Samples were then placed in 20 ml Chromacol headspace sample vials (20-HSV T229) and sealed with Chromacol 18 mm Magnetic Screw Caps with a 1 mm Silicone/PTFE Liner — Not Prefitted (18-MSC-ST101) for 6-10 days at room temperature to allow volatile organic compounds (VOCs) to accumulate in the headspace. The laboratory temperature was monitored and the average value was found to be 25 °C over a 6-month period. HS-SPME-GC/MS analysis was performed at room temperature using a DVB/CAR/PDMS SPME fibre (50/30 μm) (Supelco, 57298-U). Analysis time for the samples was 1 h and for the standard solutions was 20 s.

For the analysis of objects at Tate, a DVB/CAR/PDMS SPME fibre (50/30 μm) (Supelco, 57298-U) was placed close to the object for one week at room temperature (see Figure 1 in the main paper).

Analytes were recovered from the fibre by heating in the injection port of a Perkin Elmer Clarus 500 gas chromatograph equipped with Combipal PAL System (CTC Analytics) autosampler coupled to a Perkin Elmer Clarus 560D mass spectrometer. A VOCOL column (Supelco, 20% phenyl–80% methylpolysiloxane) was used (60 m in length and 0.25 mm in diameter) to separate the VOCs using the oven programme as follows: initial temperature of 50 °C (hold for 5 min), ramp rate of 10 °C/min to 100 °C, then 5 °C/min to 200 °C, then 2 °C/min to 220 °C, which was held for 20 min. The carrier gas was helium with a constant flow of 1 cm^3/min. The injector temperature was 250 °C and the injector was used in splitless mode with a 1 min injection. The interface and source temperatures were 200 °C and 180 °C respectively. Mass spectra were collected under electron ionisation (EI) mode at 70 eV and recorded from m/z 45–300 with a scan time of 0.4 s and an interscan delay of 0.05 s. VOC peak identification was performed using the NIST 2005 Mass Spectra Library V2.1.

Representative chromatograms showing detected VOC emissions from samples of two different modern polymeric objects are shown below in Figure 1.

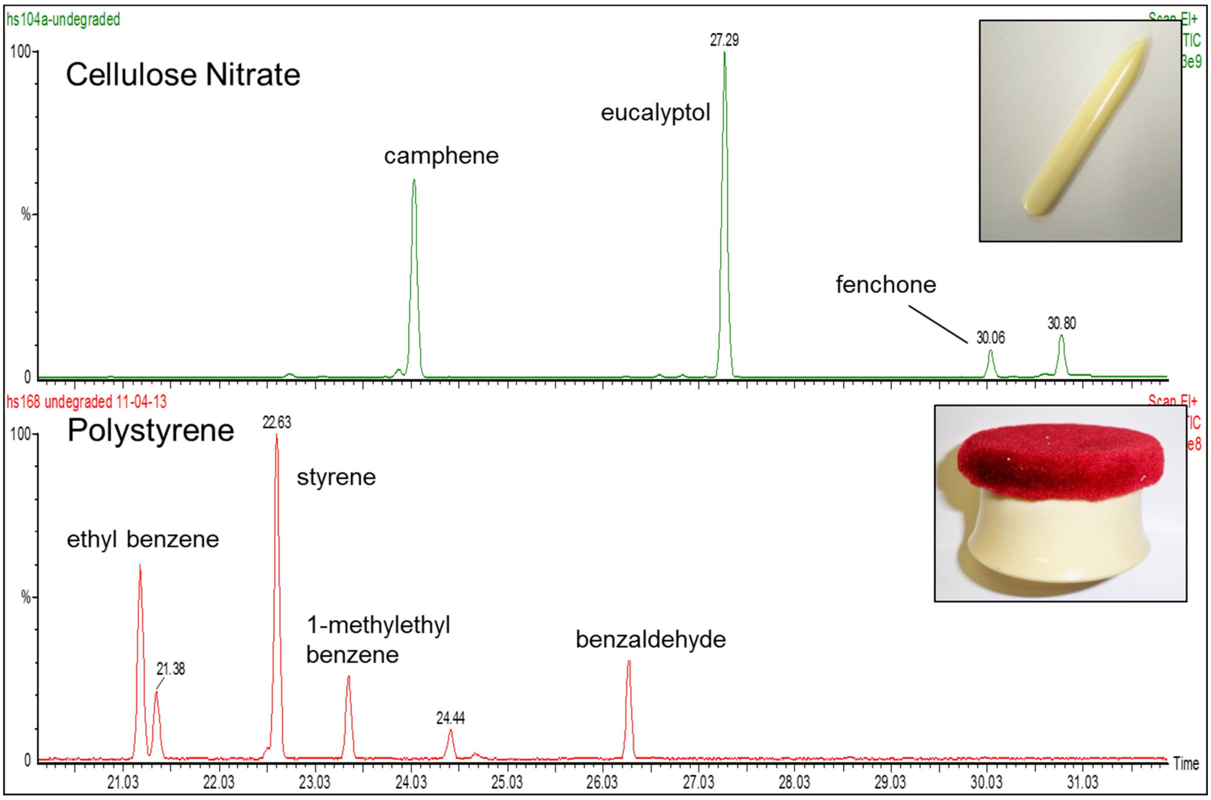

Figure 1. Chromatograms showing VOC emissions detected from samples of two different modern polymeric objects as part of this work.

3 Data processing using XCMS Online

XCMS Online is an online version of the XCMS metabolic software from the Scripps Center for Metabolomics which allows users to upload and process chromatographic data.[2] For this work, the chromatographic files generated from the GC/MS TurboMass version 6.1.0 chromatographic software were converted to .mzXML files. These were then uploaded to XCMS Online and processed. Separate jobs were run for each polymer type. Pairwise jobs were run for the samples

while Single jobs were run for the analysis of objects at Tate. The parameters used are shown in Table 2.

Table 2: Parameters used in XCMS Online for processing the chromatographic data collected from analysis of modern polymeric samples

**Parameter**	**Details**
General	
Retention time format	Minutes
Polarity	Positive
Feature Detection	
Method	centWave
ppm	100
Minimum peak width	5
Maximum peak width	10
mzdiff	0.01
Signal/Noise threshold	6
Integration method	1
Prefilter peaks	3
Prefilter intensity	100
Noise filter	0
Retention Time Correction	
Method	obiwarp
profstep	1
Alignment	
Bandwidth	10
minfrac	0.5
mzwid	0.25
minsamp	1
max	100
Statistics	
Statistical test	Unpaired parametric t-test (Welch t-test)
Perform paired test	View Pairs
Perform post-hoc analysis	True
p-value threshold (highly significant features)	0.01
Fold change	1.5
p-value threshold (significant features)	0.9
Value	into
Normalization	None
Annotation	
ppm	100
m/z absolute error	0.1
Search for	Isotopes
Identification	
ppm	100
adducts	None
Sample biosource	Default: HUMAN
Pathway ppm deviation	5
Input intensity threshold	Blank

Significant list p-value cutoff	AUTO
Visualization	
EIC width	200
Miscellaneous	
Correct mass calibration gaps	Unticked
Bypass file sanity check	Ticked

XCMS Online produced Extracted Ion Chromatograms (EICs), each of which corresponded to the detected signal across bins of m/z values of a set width.[3] Peak detection using m/z values is preferred by the developers of XCMS over detection using retention time, as it is subject to less experimental variation. Figure 2 shows an example of an EIC produced using XCMS Online, which corresponds to the detection of dimethyl phthalate from cellulose acetate and cellulose propionate samples. Multiple EICs were produced for each analyte and those that were most consistent across each sample were chosen for further analysis.

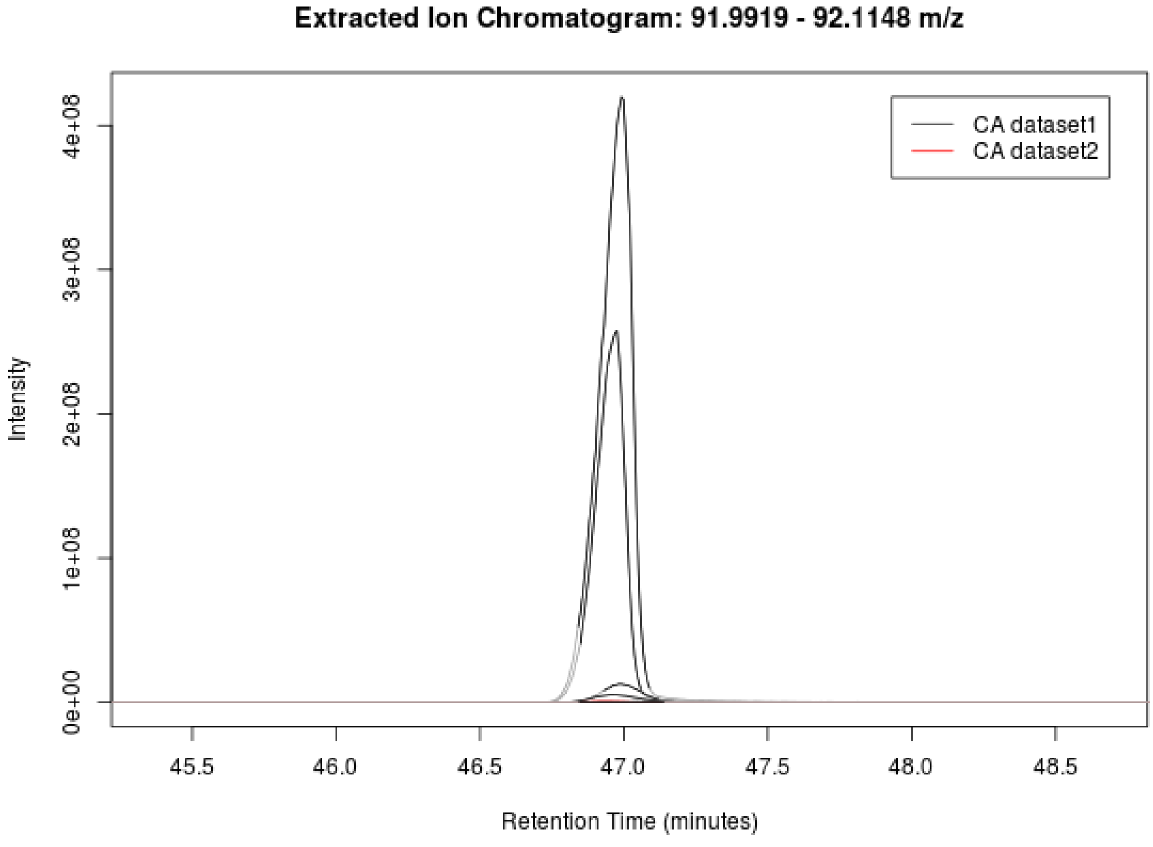

Figure 2. Extracted Ion Chromatogram produced using XCMS Online showing the signals which correspond to a retention time of approximately 47.0 mins and an m/z value of between approximately 92.0-92.1. This corresponds to dimethyl phthalate, which was detected from cellulose acetate and cellulose propionate samples.

The EICs were filtered using a second derivative Gaussian function and integrated. These were matched across different samples and retention time correction was also performed. A table of integrated peak areas and their corresponding retention times (RTs) and m/z values was then produced for each polymer type.

The chromatographic data obtained from analysis of objects at Tate was processed in the same way.

4 Microsoft Excel 2016 data processing workflow

The datatables produced from XCMS Online were transferred into Microsoft Excel 2016 for further processing. These were first placed in order of increasing RT and then filtered to remove peaks corresponding to compounds that are not of interest to this work e.g. siloxanes generated through degradation of the GC column. At each RT, multiple peaks were identified, corresponding to different m/z values found in the mass spectra. For each RT, the three most intense peaks were retained and the others were discarded.

The NIST 2005 Mass Spectra library was then used along with the TurboMass software version 6.1.0 to identify which analytes corresponded to the RTs and m/z values detected. In some cases, peaks with the same RTs and m/z values occurring in different samples were found to correspond to more than one analyte. These were discarded and only peaks which could be unambiguously identified with a single analyte were used for further analysis.

Peak areas were then weighted using external standards, according to a published method.[1] This was done by using SPME-GC/MS to analyse standard solutions on each day on which the analysis of modern polymeric samples took place, with one standard solution analysed after every six samples. This allowed interday variation in instrument performance or environmental conditions to be taken into account. The solution used was the MISA Group 17 Non-Halogen Organic Mix certified reference material, with 2000 µg/mL of each component in methanol, diluted 1/50 in methanol. Peak areas from analysis of the standard solution were used to weight the peak areas of the analytes from the modern polymeric samples run on the same day.

The natural log of each peak area was then calculated so that significant differences in low intensity peaks could be more clearly identified. Peak areas were then normalised to have a mean of 0 and a standard deviation of 1, so that different analytes could be more easily compared. This leads to some peak areas having negative values.

The data processing workflow that was performed in Microsoft Excel 2016 is summarised in Figure 3.

The data produced from the analysis of artefacts at Tate was processed in the same way.

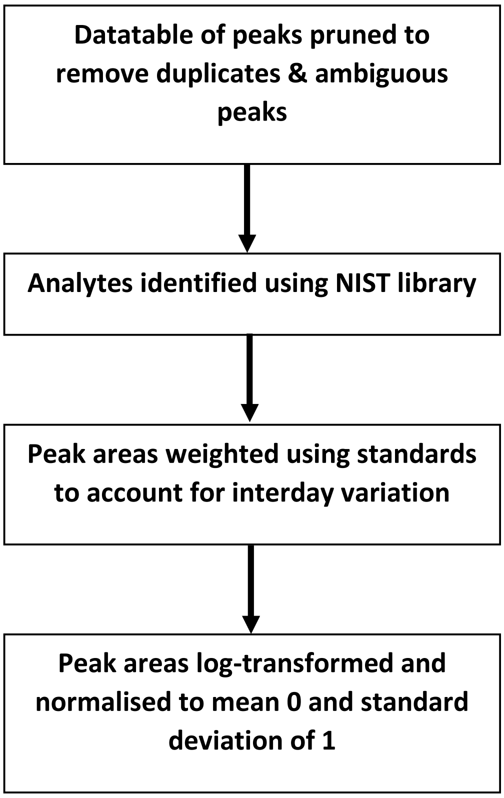

Figure 3. Workflow showing the data processing steps applied in Microsoft Excel 2016 to the datatables produced for each polymer type in XCMS Online.

5 Linear Discriminant Analysis in IBM SPSS Statistics 22

The datatables for each polymer type generated using Microsoft Excel 2016 were transferred into IBM SPSS Statistics software version 22 for Linear Discriminant Analysis (LDA). An additional column was added so that samples could be classified as described in the main paper:

- Class 1: samples artificially degraded for 0-4 weeks
- Class 2: samples artificially degraded for 6-10 weeks

The class number was used as the Grouping Variable. A further additional column was added so that a test set of samples could be identified for model validation. This was used as the Selection Variable. A value of 1 was assigned to the training set of samples while a value of 2 was assigned to the test set of samples. The parameters used for Linear Discriminant Analysis are shown in Table 3.

Table 3: Parameters used in IBM SPSS Statistics 22 for Linear Discriminant Analysis.

Parameter	Details
Statistics	
Descriptives	All ticked
Function Coefficients	Fisher's
Matrices	All unticked
Classify	
Prior probabilities	Compute from group sizes

Display	All ticked
Use Covariance Matrix	Within-groups
Plots	Combined-groups plot
Replace missing values with mean	Unticked
Save	
Predicted group membership	Unticked
Discriminant scores	Unticked
Probabilities of group membership	Unticked
Export model information to XML file	No file selected
Bootstrap	
Perform bootstrapping	Unticked

In an initial analysis, all detected VOCs were used as Independent Variables. Using the Tests of Equality of Group Means, the VOCs with the most significant differences in their peak areas between Class 1 and Class 2 were identified. Different combinations of these VOCs were then used as independent variables for further LDA. The combination of VOCs that produced the most accurate classifications of test sets of samples (validation) into the two classes described above are reported in the main paper.

An example of the outputs from LDA using IBM SPSS Statistics software version 22 is shown in Table 4. These results were obtained from LDA of cellulose propionate (CP) samples using dimethyl phthalate and propanoic acid as the independent variables. As can be see, the test set contained 13 samples of which 7 belonged to group 1 (Class 1, the less degraded samples) and 6 belonged to group 2 (Class 2, the more degraded samples). Of the samples in Class 1, 5 out of 7 were accurately classified, while 5 out of 6 samples from Class 2 were classified accurately. The classification accuracy is thus 77% as shown in footnote (a) in Table 4.

Validation accuracy is shown in the section marked "Cases Not Selected", indicating that the samples classified in this approach were separate from those used to develop the model. The test set contained 6 samples, of which 3 samples belonged to Class 1 and 3 to Class 2. Accurate classifications were obtained for 2 out of 3 samples from Class 1 while all samples in Class 2 were accurately classified. The validation accuracy is thus 83% as shown in footnote (b) in Table 4. Cross-validation was also performed, this involves removing a single sample from the training set and then using the obtained model to classify that sample. This is repeated for all samples. The cross-validation accuracy of the analysis shown below is 77% (footnote (d) in Table 4). The cross-validation accuracies are not reported in the main paper as validation using a separate test set is more robust.

Table 4. Sample results from a single linear discriminant analysis of cellulose propionate samples. The validation accuracy is 83%

Classification Results[a,b,d]

			Degraded	Predicted Group Membership 1.00	2.00	Total
Cases Selected	Original	Count	1.00	5	2	7
			2.00	1	5	6
		%	1.00	71.4	28.6	100.0
			2.00	16.7	83.3	100.0
	Cross-validated[c]	Count	1.00	5	2	7
			2.00	1	5	6
		%	1.00	71.4	28.6	100.0
			2.00	16.7	83.3	100.0
Cases Not Selected	Original	Count	1.00	2	1	3
			2.00	0	3	3
			Ungrouped cases	11	1	12
		%	1.00	66.7	33.3	100.0
			2.00	.0	100.0	100.0
			Ungrouped cases	91.7	8.3	100.0

a. 76.9% of selected original grouped cases correctly classified.

b. 83.3% of unselected original grouped cases correctly classified.

c. Cross validation is done only for those cases in the analysis. In cross validation, each case is classified by the functions derived from all cases other than that case.

d. 76.9% of selected cross-validated grouped cases correctly classified.

A table such as that shown in Table 4 was produced for each validation using a different test set. Between 3-6 test sets were used for each polymer type and the validations were tested for multiple combination of VOCs. The average of the validation accuracies achieved using different test sets for a particular combination of VOCs was used to assess which combination of VOCs gave the most accurate overall classifications.

Classification of the artefacts from Tate based on their VOC emissions was performed in the same way, however ratios of VOCs rather than peak areas were used as explained in the main paper. This was to allow for differences in object mass and the volume of the headspace analysed.

6 Data from analysis of VOC emissions from modern polymeric samples

6.1 Data from analysis of VOC emissions from Cellulose propionate

The VOCs that were found to give the most accurate validations for cellulose propionate (CP) samples were propanoic acid and dimethyl phthalate. The results of LDA for these samples is summarised in Table 5. The data in the third row of results corresponds to the results shown in Table 4.

Table 5. Summarised results of Linear Discriminant Analysis of cellulose propionate samples

VOCs	No. of samples used to build model	Test Set	Classification Accuracy	Validation Accuracy
Propanoic acid, Dimethyl phthalate	13	HS34[a]	85	67
Propanoic acid, Dimethyl phthalate	13	HS35[a]	85	83
Propanoic acid, Dimethyl phthalate	13	HS45[a]	77	83
		**Average**	82	78

[a] The test set corresponds to 6 samples, each taken from the same object but degraded for different lengths of time, either 0,2,4,6,8 or 10 weeks at 80 °C and 65% RH.

The average classification accuracy was found to be 82% and the average validation accuracy was 78%. These figures are reported in the main paper.

It should be noted that 5 samples of cellulose acetate (CA) were also analysed using SPME-GC/MS analysis and the VOC data was processed in the same way as that for the CP samples. However, there were not enough artificially degraded samples to explore classification. Attempts at classifying the CA and CP samples as a grouped set were less successful than those for the CP samples on their own.

6.2 Data from analysis of VOC emissions from Cellulose nitrate

The VOCs that were found to give the most accurate validations for cellulose nitrate (CN) samples were furfural, a terpene identified as either 3-carene or β-terpinene, camphene and campholenal. The results of LDA for these samples is summarised in Table 6.

Table 6. Summarised results of Linear Discriminant Analysis of cellulose nitrate samples

VOCs	No. of samples used to build model	Test Set	Classification Accuracy	Validation Accuracy
Furfural, terpene1, camphene, campholenal	19	HS104[a]	90	83
Furfural, terpene1, camphene, campholenal	19	HS3[a]	100	83
Furfural, terpene1, camphene, campholenal	19	HS2[a]	90	83
		**Average**	93	83

[a] The test set corresponds to 6 samples, each taken from the same object but degraded for different lengths of time, either 0,2,4,6,8 or 10 weeks at 80 °C and 65% RH.

The average classification accuracy was found to be 93% and the average validation accuracy was 83%. These figures are reported in the main paper.

6.3 Data from analysis of VOC emissions from Polyethylene

The VOCs that were found to give the most accurate validations for polyethylene (PE) samples were decane and camphor. The results of LDA for these samples is summarised in Table 7.

Table 7. Summarised results of Linear Discriminant Analysis of polyethylene samples

VOCs	No. of samples used to build model	Test Set	Classification Accuracy	Validation Accuracy
Decane, camphor	42	HS13[a]	62	50
Decane, camphor	42	HS24[a]	60	50
Decane, camphor	42	HS28[a]	67	50
Decane, camphor	42	HS31[a]	67	50
Decane, camphor	42	HS56[a]	62	67
Decane, camphor	42	HS57[a]	62	50
		**Average**	63	53

[a] The test set corresponds to 6 samples, each taken from the same object but degraded for different lengths of time, either 0,2,4,6,8 or 10 weeks at 80 °C and 65% RH.

The average classification accuracy was found to be 63% and the average validation accuracy was 53%. These figures are reported in the main paper.

6.4 Data from analysis of VOC emissions from Polystyrene

The VOCs that were found to give the most accurate validations for polystyrene (PS) samples were cis-β-methylstyrene, acetophenone and (1-methylethyl)-benzene. The results of LDA for these samples is summarised in Table 8.

Table 8. Summarised results of Linear Discriminant Analysis of polystyrene samples

VOCs	No. of samples used to build model	Test Set	Classification Accuracy	Validation Accuracy
cis-β-methylstyrene, acetophenone, (1-methylethyl)-benzene	24	HS36[a]	83	33
cis-β-methylstyrene, acetophenone, (1-methylethyl)-benzene	24	HS37[a]	83	50
cis-β-methylstyrene, acetophenone, (1-methylethyl)-benzene	24	HS14[a]	71	83
cis-β-methylstyrene, acetophenone, (1-methylethyl)-benzene	24	HS38, HS4, HS118, HS141, HS145[b]	72	80
		**Average**	77	62

[a] The test set corresponds to 6 samples, each taken from the same object but degraded for different lengths of time, either 0,2,4,6,8 or 10 weeks at 80 °C and 65% RH.

[b] This test set corresponds to samples taken from different objects, with one sample being taken from each.

The average classification accuracy was found to be 77% and the average validation accuracy was 62%. These figures are reported in the main paper.

6.5 Data from analysis of VOC emissions from Polyurethane

The VOCs that were found to give the most accurate validations for polyurethane (PUR) samples were camphor, phenol, pentanal, 3,5-dimethyloctane and styrene . The results of LDA for these samples is summarised in Table 9.

Table 9. Summarised results of Linear Discriminant Analysis of polyurethane samples

VOCs	No. of samples used to build model	Test Set	Classification Accuracy	Validation Accuracy
camphor, phenol, pentanal, 3,5-dimethyloctane, styrene	30	HS86[a]	87	83
camphor, phenol, pentanal, 3,5-dimethyloctane, styrene	30	HS89[a]	87	67
camphor, phenol, pentanal, 3,5-dimethyloctane, styrene	30	HS90[a]	90	83
camphor, phenol, pentanal, 3,5-dimethyloctane, styrene	30	HS85[a]	83	83
		**Average**	87	79

[a] The test set corresponds to 6 samples, each taken from the same object but degraded for different lengths of time, either 0,2,4,6,8 or 10 weeks at 80 °C and 65% RH.

The average classification accuracy was found to be 87% and the average validation accuracy was 79%. These figures are reported in the main paper.

6.6 Data from analysis of VOC emissions from Poly(vinyl chloride)

The VOCs that were found to give the most accurate validations for poly(vinyl chloride) (PVC) samples were hexanal, 2-ethylhexanol and limonene. The results of LDA for these samples is summarised in Table 10.

Table 10. Summarised results of Linear Discriminant Analysis of poly(vinyl chloride) samples

VOCs	No. of samples used to build model	Test Set	Classification Accuracy	Validation Accuracy
hexanal, 2-ethylhexanol, limonene	33	HS33[a]	82	50
hexanal, 2-ethylhexanol, limonene	33	HS61[a]	82	50
hexanal, 2-ethylhexanol, limonene	33	HS62[a]	82	50
		**Average**	82	50

[a] The test set corresponds to 6 samples, each taken from the same object but degraded for different lengths of time, either 0,2,4,6,8 or 10 weeks at 80 °C and 65% RH.

The average classification accuracy was found to be 82% and the average validation accuracy was 50%. These figures are reported in the main paper.

7. Analysis at Tate

Results from analysis at Tate are shown below. In the case of the CA objects, the ratio of acetic acid to dimethyl phthalate was used and compared with the ratio of propanoic acid to dimethyl phthalate from the CP samples analysed in the earlier part of the research. A comparison of these ratios can be shown in Figure 4.

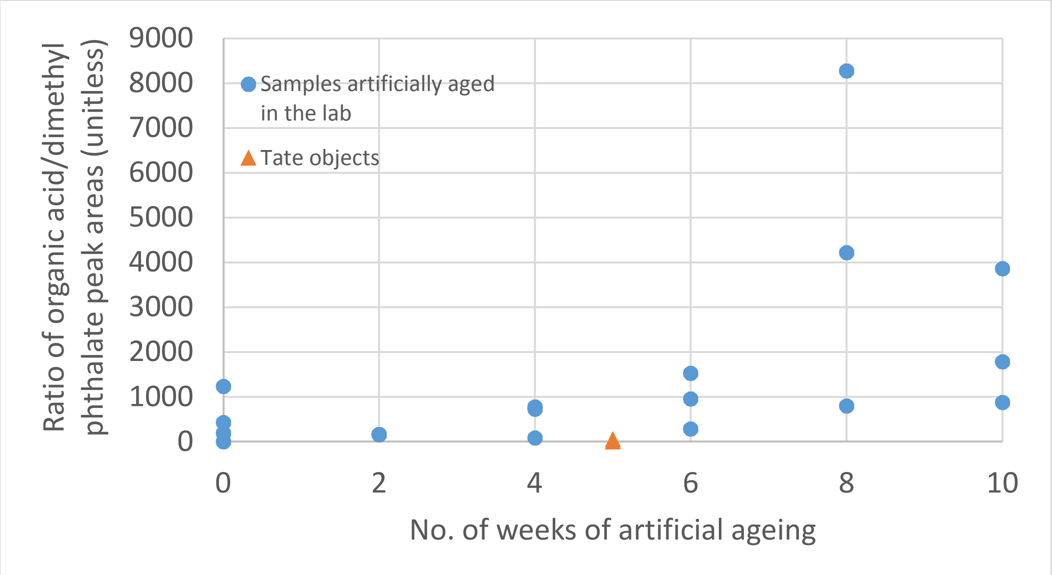

Figure 4. Ratio of organic acid/dimethyl phthalate peak areas from artificially aged samples and from the objects at Tate. The x-values from the Tate object are arbitrarily placed at the centre of the x-axis, as they do not correspond to a particular number of weeks of artificial ageing.

This data shows that the ratio for both objects from Tate is low, more similar to those objects degraded for 0-4 weeks than for 6-8 weeks, indicating lower levels of organic acid emissions and higher levels of plasticiser content.

Using a classification based on Linear Discriminant Analysis and the ratios of the relevant peak areas, performed as described above, both Tate objects were classified as being part of Class 1.

8 Bibliography

[1] K. Curran, M. Underhill, L. T. Gibson, and M. Strlič, *Microchem. J.* **2016**, *124*, 909.

[2] R. Tautenhahn, G. J. Patti, D. Rinehart, and G. Siuzdak, *Anal. Chem.* **2012**, *84*, 5035

[3] C. A. Smith, E. J. Want, G. O'Maille, R. Abagyan, and G. Siuzdak, *Anal. Chem.* **2006**, *78*, 779.
